# Preserved Tool Knowledge in the Context of Impaired Action Knowledge: Implications for Models of Semantic Memory

**DOI:** 10.3389/fnhum.2013.00120

**Published:** 2013-04-29

**Authors:** Frank E. Garcea, Mary Dombovy, Bradford Z. Mahon

**Affiliations:** ^1^Department of Brain and Cognitive Sciences, University of RochesterRochester, NY, USA; ^2^Department of Rehabilitation and Neurology, Unity HospitalRochester, NY, USA; ^3^Department of Neurosurgery, University of RochesterRochester, NY, USA; ^4^Center for Visual Science, University of RochesterRochester, NY, USA

**Keywords:** embodied cognition, cognitive neuropsychology, concepts, action recognition, action production, tools

## Abstract

A number of studies have observed that the motor system is activated when processing the semantics of manipulable objects. Such phenomena have been taken as evidence that simulation over motor representations is a necessary and intermediary step in the process of conceptual understanding. Cognitive neuropsychological evaluations of patients with impairments for action knowledge permit a direct test of the necessity of motor simulation in conceptual processing. Here, we report the performance of a 47-year-old male individual (Case AA) and six age-matched control participants on a number of tests probing action and object knowledge. Case AA had a large left-hemisphere frontal-parietal lesion and hemiplegia affecting his right arm and leg. Case AA presented with impairments for object-associated action production, and his conceptual knowledge of actions was severely impaired. In contrast, his knowledge of objects such as tools and other manipulable objects was largely preserved. The dissociation between action and object knowledge is difficult to reconcile with strong forms of the embodied cognition hypothesis. We suggest that these, and other similar findings, point to the need to develop tractable hypotheses about the dynamics of information exchange among sensory, motor and conceptual processes.

## Introduction

On a daily basis we do remarkable things: we drive our automobiles to work, we send messages to our friends with the push of a few buttons, and use tools that extend the capabilities of our bodies. An indefinite set of object concepts are spontaneously called upon in the service of our day-to-day interactions with the environment. How are object concepts organized and represented in such a way to make everyday behavior possible? How do sensory and motor representations contribute to the organization and representation of object concepts? A prominent theory that proposes an answer to these questions is the embodied cognition hypothesis. That hypothesis argues that conceptual knowledge consists, in whole or in part, in the simulation, or re-enactment of the same sensorimotor processes that are engaged during actual interactions with the relevant types of stimuli. The first clear articulation of this proposal was by Allport (1985):

“The essential idea is that the same neural elements that are involved in coding the sensory attributes of a (possibly unknown) object presented to the eye or hand or ear also make up the elements of the auto-associated activity-patterns that represent familiar object concepts in ‘semantic memory.’ This model is, of course, in radical opposition to the view, apparently held by many psychologists, that ‘semantic memory’ is represented in some abstract, modality-independent, ‘conceptual’ domain remote from the mechanisms of perception and of motor organization.” (p. 53).

On that hypothesis, when one is asked to name a hammer, a necessary, and intermediary step in the naming process involves retrieval of motor-relevant information associated with the use of hammers (e.g., Barsalou, 1999, 2008; Glenberg and Kaschak, 2002; Barsalou et al., 2003; Simmons and Barsalou, 2003; Zwaan, 2004; Gallese and Lakoff, 2005; Pulvermüller, 2005; Kiefer and Pulvermüller, 2012). The embodied cognition hypothesis thus predicts that if an individual were to incur brain injury that impaired his/her ability to use tools, then the person would also have a conceptual impairment for tools. In Allport’s (1985) words: “… the loss of particular attribute information in semantic memory should be accompanied by a corresponding *perceptual* (agnostic) deficit.” (1985, p. 55; emphasis in original). In other words, according to the embodied cognition hypothesis of tool recognition, loss of motor knowledge about how to use tools should be associated (necessarily) with a corresponding semantic deficit. This prediction can be tested with cognitive neuropsychological evaluations of individuals with acquired brain damage. The goal of the current investigation was to test the embodied cognition hypothesis of tool recognition with a detailed case study of a 47-year-old individual who sustained a left cerebrovascular accident (CVA) and presented with a circumscribed impairment for knowledge of the typical actions associated with objects.

### Empirical and theoretical background

The embodied cognition hypothesis of concept representation is an example of a broader theoretical framework based on the idea that comprehension involves covert production. Perhaps the best known example of this class of theories is the motor theory of speech perception (e.g., Liberman et al., 1967; Liberman and Mattingly, 1985; for a recent review, see Galantucci et al., 2006). That theory made the important contribution of emphasizing the idea that recognition should not be conceived of as a passive process of, for instance, matching a percept to a template stored in memory. Motor theories of perception have recently gained widespread popularity in the context of the putative mirror properties of some neurons in premotor and parietal regions of the macaque. In macaques, it has been shown that neurons in premotor and parietal cortex are activated when performing gestures and when observing others perform gestures (i.e., mirror neurons). This finding has been argued to provide support for the hypothesis that motor processes involved in action production are constitutively (i.e., necessarily) involved in action recognition (di Pellegrino et al., 1992; Gallese et al., 1996; Rizzolatti et al., 2001; for review see Rizzolatti and Arbib, 1998; Rizzolatti and Craighero, 2004; Rizzolatti and Sinigaglia, 2010) for critical reviews and discussion see Mahon and Caramazza, 2005; Dinstein et al., 2008; Hickok, 2009, 2010; Stasenko et al., in press).

However, whereas motor theories of action recognition are proposals about how perceptual information is comprehended and interpreted, the embodied hypothesis of concept representation is a claim about the representation of object concepts. A range of findings has been argued to support the embodied cognition hypothesis of concept representation. For instance, it has been shown that transcranial magnetic stimulation (TMS) of somatotopic specific portions of motor cortex selectively affects processing of information relevant to the corresponding effector (words describing hand actions, or foot actions; Pulvermüller et al., 2005; for review see Pulvermüller, 2005). Another TMS-based finding is that there is modulation of motor-evoked potentials (MEPs) in distal limb muscles associated with corresponding effector-specific action words. For instance, MEPs in hand muscles are modulated by processing of hand-related action words compared to foot-related action words (Buccino et al., 2005; Papeo et al., 2009). In sum, data from TMS have shown that there is an association between the activation of the motor system and comprehension of action words, in a somatotopic manner. That basic phenomenon has also been observed using functional magnetic resonance imaging (fMRI; Buccino et al., 2001; Hauk et al., 2004; Tettamanti et al., 2005).

Another class of findings demonstrates automatic activation of object use information when viewing manipulable objects. A widely replicated finding is differential BOLD contrast in parietal and premotor structures when naming or viewing tools (e.g., Chao and Martin, 2000; Noppeney et al., 2006; Mahon et al., 2007). These data have been taken as evidence for the automatic retrieval of motor-relevant information associated with the processing of tools. Finally, a number of behavioral findings have also been argued to support the claim that the motor system is involved in language comprehension. The most common finding is that response times (RTs) are facilitated when processing the semantics of sentences whose meaning implies an action in the same direction as a manual response (toward the body; away from the body; e.g., the “Action-sentence Compatibility Effect,” or ACE, of Glenberg and Kaschak, 2002; Glenberg et al., 2008).

### The current investigation

If conceptual understanding of tools and their names necessarily involves simulation of motor-relevant content, it follows that impairments affecting knowledge of object-associated actions should be associated with conceptual impairments for tools. To foreshadow the results, Case AA presented with an action production impairment (i.e., apraxia of object use), as well as an impairment for conceptual knowledge of actions. However, his ability to extract semantic information from object stimuli remained relatively intact. The results are discussed in the context of the embodied cognition hypothesis and alternative explanations of the empirical phenomena that have been argued to support that theory.

## Case Report

**Figure 1:**
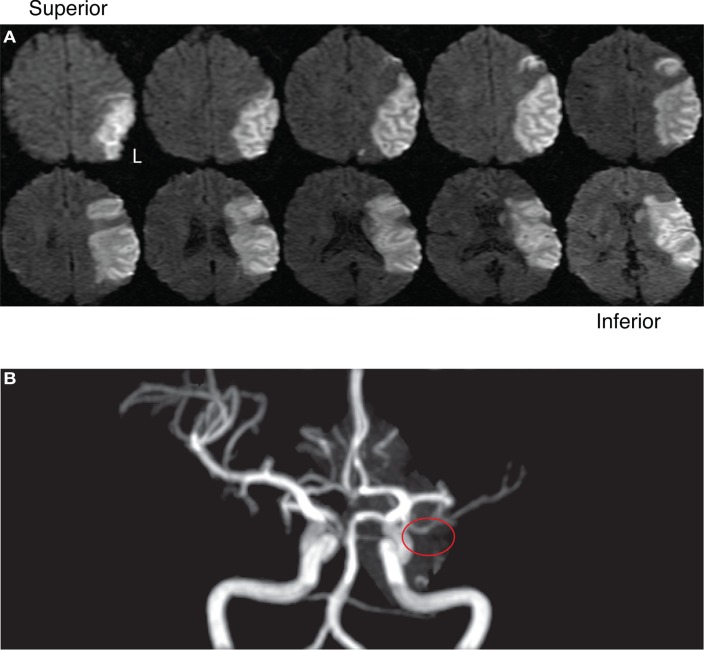
**(A)** Diffusion-weighted images of Case AA’s left-hemisphere lesion. **(B)** Angiography and origin of Case AA’s left-hemisphere lesion.

Case AA was a right-handed man born in 1963 with 13 years of education who suffered an ischemic stroke in February 2010. Diffusion-weighted images taken at the time of clinical care in February 2010 revealed a large left-sided infarction (see Figure 1A); the occlusion originated in the distal M1 branch of the left middle cerebral artery (MCA), sparing the anterior and posterior cerebral arteries (see Figure 1B). Case AA’s ischemic stroke lesioned a large portion of frontal and parietal cortex, pre/post-central gyrus, and posterior lateral temporal cortex. We first saw this individual in February 2011 when he was referred from the Unity Rehabilitation and Neurology Center in Greece, NY, USA; he had hemiplegia that affected the mobility of his right arm and leg. His speech and executive functioning were affected by the stroke as well. All testing sessions took place between February 2011 and June 2011. Case AA gave informed written consent in accordance with the University of Rochester Institutional Review Board.

### Control participants

Six participants (males) served as controls for Case AA’s performance. All control participants gave written informed consent in accordance with the University of Rochester Institutional Review Board. Control participants had no history of neurological illness, and were matched to Case AA for age (mean = 49.3 years; range 42–55 years), education level (mean = 14.9 years; range = 12–18 years), and handedness (Edinburgh Handedness Questionnaire, Oldfield, 1971; mean = 0.92; range = 0.53–1; Case AA’s reported pre-morbid handedness coefficient = 1). Control participants completed the battery of tests in two sessions that lasted approximately 2 h each. Unless otherwise noted, control performance refers to this group of matched controls.

### General methods

Across all tasks, unless otherwise noted, Case AA was asked to quickly and accurately complete every trial. Each trial lasted 10 s or until a response was given, whichever came first. If Case AA was not able to respond in 10 s the trial was considered incorrect and scored as zero. All picture stimuli were grayscale and 400 by 400 pixels (all in-house test stimuli can be found in the Supplementary Material). For experiments requiring overt verbal responses, responses were spoken into a microphone and stimulus presentation, and response recordings were controlled with DMDX (Forster and Forster, 2003). The responses were analyzed offline as wav files. All experiments that required keyboard presses were controlled with EPrime Software 2.0 (Psychology Software Tools, Pittsburgh, PA, USA). (Monitor information: View Sonic, 1620 × 1050 pixels, 120 Hz).

### Statistical analyses

Modified *t*-tests were computed to assess if the performance of Case AA was different from the performance of the control participants using software provided by Crawford et al. (1998) and Crawford et al. (2010)^1^.The software takes as input healthy control participants’ mean, standard deviation, number of control participants, and the patient’s score, and computes a *t*-test, a point interval (percentage of the population that would have a lower score), 95% confidence intervals associated with the point interval, an effect size (*z*-score) associated with the patient’s performance, and 95% confidence intervals on the effect size^2^.

The Revised Standardized Difference Test (RSDT) was used to calculate a dissociation between Case AA’s performance on two tests. The RSDT takes as input the patient’s performance on two tests, as well as control participants’ mean, standard deviation, and the correlation between control participants’ scores on the two tests. The program computes the same measurements as above, and tests whether the patient’s accuracy difference between two tests meets the criterion for a dissociation (strong or classical; for precedent, see Shallice, 1988); dissociations may be “classical” (Case AA is impaired on Task 1 but not on Task 2) or “strong” (Case AA is impaired on Task 1 and Task 2, but Task 1 is impaired to a greater degree than Task 2).

## Neuropsychological Evaluation

### Experimental study I: Visual object recognition, linguistic processing, and visual long-term memory encoding

Case AA was administered a battery of tests probing mid- and high-level visual processing, number identification, word reading, short-term memory retrieval, and visual long-term memory encoding and retrieval. Here we give a brief overview of his (generally intact) performance (for details, see the Methods and Results in the Supplementary Materials).

#### Visual object recognition

Case AA’s motion and color perception, object decision, and letter identification were within control range or at ceiling (see Table S1A in Supplementary Material). Case AA was flawless when naming one- and two-digit numbers. He was impaired relative to controls when naming three-digit numbers (*p* < 0.05), making two errors mixing the order of the digits, Case AA had a mild impairment when asked to match two of three overlapping figures (*p* < 0.05). Case AA’s performance on the Birmingham Object Recognition Battery (BORB; Riddoch and Humphreys, 1993) was within the range of controls on all the subtests he completed (See Table S1A in Supplementary Material for all results).

#### Linguistic processing: the psycholinguistic assessment of language processing in Aphasia

Case AA was similar to controls across a number of The Psycholinguistic Assessment of Language Processing in Aphasia (PALPA; Kay et al., 1992) word reading tests that manipulated various psycholinguistic properties of words (e.g., imageability, frequency, grammatical class, spelling irregularity, etc., see Table S2A in Supplementary Material). The only difficulty Case AA had was with reading non-words with four letters (3/6, 50%; *p* < 0.05), and reading low imageability and low frequency words (18/20, 90%; *p* < 0.01). Independent of those factors, his ability to read words from different grammatical classes (nouns, verbs, adjectives) was comparable to controls (see Table S2A in Supplementary Material for all results).

#### Sentence repetition

Case AA successfully repeated 34 out of 36 sentences auditorily presented by the experimenter (FG). Of the two errors that Case AA committed, both involved rearranging one word in an auditorily presented sentence, and pluralizing one word,

Experimenter: “The horse’s got less chickens to scare.”

Case AA: “The horse’s got more chickens to scare.”

Experimenter: “The man’s moving the horse.”

Case AA: “The man’s moving with horses.”

#### Cookie theft

Case AA’s spontaneous language production was evaluated several times with the Cookie Theft test, a subtest of the Boston Diagnostic Aphasia Examination (BDAE; Goodglass and Kaplan, 1972). Case AA was given 2 min to provide as detailed a description as possible. Generally, across all testing sessions Case AA’s speech was fluent but clearly impoverished. He did not make phonological or morphological errors when explaining the contents of the scene.

2.14.2011. They’re standing on a cookie jar and uh, he’s falling. She’s washing dishes, the sink is overflowing with water.

2.23.2011. She’s reaching for the cookie jar, up on the stool, the stool’s about to fall over. She’s washing dishes, but the dishes are overflowing, going onto the floor. She’s laughing.

#### Visual long-term memory encoding and retrieval

Case AA’s ability to encode long-term semantic information from visually presented stimuli was also within control range; when asked to identify repeated images embedded within a series of 216 images, Case AA was at ceiling (task and stimuli modified from Brady et al., 2008). All results can be found in Table S3 in Supplementary Material.

### Discussion

Case AA performed within control range or had only mild impairments on a number of tasks investigating visual perception, visual object recognition, long-term visual memory, word and number reading, and spontaneous speech. His ability to follow directions and perform various tasks was not affected by his brain injury. Having ruled out general impairments Case AA may have had with object recognition, language, and memory, and ensuring his ability to follow directions over different forms of input and output was intact, we set out to characterize the boundaries of Case AA’s impairment for action knowledge, specifically at the semantic level.

### Experimental study II: Action production and action recognition

#### Action recognition: action decision

Two videos of an individual (FG) performing actions were presented for Case AA on every trial, and he had to decide which was meaningful/real. Real actions (e.g., intransitive: saluting) were gestures that conveyed meaning, while “unreal” actions were gestures that did not convey meaning but made similar use of the limbs. Case AA was at ceiling when making action reality decisions over meaningful intransitive action clips (10/10).

#### Pantomime discrimination

**Figure 2:**
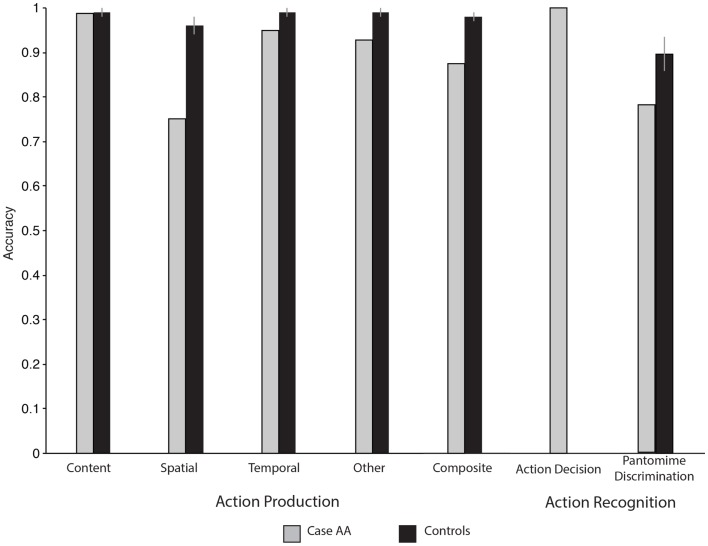
**The dissociation between Case AA’s ability to produce meaningful actions and Case AA’s ability to recognize meaningful action**.

Eighteen videos of transitive actions were centrally presented with two words denoting objects to the left and to the right below the video. On every trial Case AA was asked to decide which object was used in the action being pantomimed in the video. Case AA was not significantly impaired relative to controls for discriminating pantomimes (14/18, 78%, *p* = 0.22). See Table 1 for all Action Recognition results; see also Figure 2.

**Table 1:** **Action recognition**.

Action Recognition	Control sample			Case AA’s score	Significance test	
	*n*	Mean	SD		*t*	*p*
Action decision	–	–	–	1	–	–
Pantomime discrimination	6	0.9	0.08	0.78	−1.39	0.22

*Control participants (*n*), mean control proportion correct (Mean), control standard deviation (SD), Case AA’s proportion correct (Case AA’s scores) and *t*- and *p*-scores characterizing the difference between Case AA and control participants*.

#### Action production: overview of methods and tasks

Over multiple sessions Case AA was asked to imitate transitive and intransitive pantomimes, to pantomime transitive and intransitive actions from verbal command, and tactilely identify and use objects in hand. Because Case AA had a right hemiplegia, he was confined to using his non-dominant left hand for all action production tasks; thus, all control participants used their non-dominant left hand when performing actions. Fifteen objects (hammer, screwdriver, scissors, hairbrush, spray bottle, spoon, cup, pliers, wrench, stapler, hole puncher, nail clipper, paint roller, feather duster, clothespin) were used across multiple tests probing action and object knowledge; 10 gestures that did not necessitate the use of objects were also used (i.e., intransitive actions: peace sign, thumbs up, hitchhiking, waving goodbye, beckoning “come here,” making a fist, military salute, gesturing crazy, signaling someone to stop, signaling to be quiet). For all action production tasks (pantomime from verbal command, imitation), pantomimes were blocked by type (e.g., transitive/intransitive) and Case AA was asked to perform each pantomime immediately after the experimenter had completed the action; if Case AA was not able to respond within 10 s the trial was scored as a zero. However, if Case AA responded within 10 s, he was given ample time to produce the action. For the pantomime imitation tasks, the experimenter (FG) performed a transitive or intransitive gesture on each trial and Case AA was asked to imitate the gesture immediately after the experimenter had completed the action. If Case AA did not imitate within 10 s after the experimenter finished the action the trial was scored as a zero.

All actions, for both Case AA and controls, were scored using the criteria established by Power et al. (2010). The Florida Apraxia Battery-Extended and Revised Sydney (FABERS) is set of scoring criteria for apraxia that accounts for the diverse types of apraxic errors. The scoring criteria are organized by content errors (e.g., perseverations, semantically related responses), spatial errors (e.g., misconfigurations of fingers/limb, body part as tool), temporal errors (e.g., incorrect sequencing of actions), and “other” errors (e.g., incorrect pantomime not used in test, failure to produce any response). This scoring approach thus registers the specific error patterns of patients while accounting for healthy performance for other aspects of the action.

Case AA and control participants’ actions were video recorded and scored offline by the experimenter (FG) and an individual naïve to the goal of the current investigation. For each trial, the video was scored for each dimension as specified in the FABERS protocol. For instance, there are several types of content errors that apraxics may commit (e.g., semantically related errors such as pantomiming the use of a hammer when asked to pantomime using a butcher knife), or several types of spatial errors apraxics commit (e.g., using their hands/fingers to pantomime object use (body-part-as-tool – BPAT – errors) or internal/external configuration errors that index abnormal hand/arm posture with respect to how the object should be appropriately manipulated). For a description of the error types see Appendix F from Power et al., 2010; for precedent see Rothi et al. (1988, 1997).

The experimenter (FG) and a naïve individual coded every action along the 15 dimensions (i.e., Case AA and controls were given a “1” if the action was in accordance with each individual dimension, or “0” if the action was incorrect along the various dimensions). If Case AA and controls accurately produced an action, they received a score of 15 for that action. In the situation where Case AA sporadically would forget how to pantomime an object’s use (which is scored in the ‘other’ error type), his action was not coded “0” for content, spatial, and temporal errors (i.e., actions were only coded as errors that Case AA and controls committed). In this way, failure to produce an action effectively removed that item from the analysis of the error types, in order to have a “clean” measure of his error breakdown by type. When calculating Case AA’s performance along content, spatial, temporal, and “other,” the final score was derived by averaging within error type, across objects, which resulted in a vector of 15 values (one for every error type) for each coder; coder values were then averaged. In order to measure Case AA’s object use, values within object, collapsing across error type, were averaged for each coder; this resulted in a vector of 15 values (one for every object) for each coder; coder object values were then averaged for each object, and the average of all object values were then averaged together to derive the object use metric. This scoring protocol was carried out for Case AA and control participants.

#### Pantomime from verbal command: transitive actions

A composite score for overall object use can be derived by averaging across all error types for each action; Case AA was impaired with respect to control participants (13.1/15, 87%, *p* < 0.001; see Table 2.). The analysis by error type revealed that Case AA was normal with respect to content-related properties when pantomiming transitive actions (14.9/15, 99%, *p* = 1), but was impaired for spatial properties of the same actions (11.4/15, 76%, *p* < 0.001). The temporal aspects of Case AA’s transitive pantomimes were also (albeit more mildly), affected (14.3/15, 95%, *p* < 0.05). The final error category within the FABERS scoring system is somewhat of a catch-all (e.g., unrecognizable action production); Case AA was impaired along this dimension as well (14/15, 93%; *p* < 0.01), principally reflecting his sporadic failure to pantomime object use (see Figure 2).

**Table 2:** **Action production**.

	Control Sample			Case AA’s score	Significance test	
	*n*	Mean	SD		*t*	*p*
**PANTOMIME FROM VERBAL COMMAND:TRANSITIVE**						
Content	6	0.99	0.01	0.99	0	1.00
Spatial	6	0.98	0.02	0.76	−10.18	<0.001
Temporal	6	0.99	0.01	0.95	−2.77	0.04
Other	6	0.99	0.01	0.93	−5.56	0.003
Object use	6	0.98	0.01	0.87	−10.18	<0.001
**PANTOMIME FROM COMMAND: INTRANSITIVE**						
Content	6	1	–	1	–	–
Spatial	6	1	–	1	–	–
Temporal	6	1	–	0.98	–	–
Other	6	1	–	0.98	–	–
**PANTOMIME IMITATION: TRANSITIVE**						
Content	6	1	–	0.99	–	–
Spatial	6	0.98	0.02	0.77	−9.72	<0.001
Temporal	6	0.99	0.01	0.95	−3.70	0.01
Other	6	1	–	1	–	–
Object use	6	0.98	0.01	0.91	−6.48	<0.001
**PANTOMIME IMITATION: INTRANSITIVE**						
Content	6	1	–	1	–	–
Spatial	6	1	–	0.99	–	–
Temporal	6	1	–	0.98	–	–
Other	6	1	–	1	–	–
**TACTILE RECOGNITION, OBJECT USE, AND KNOWLEDGE OF OBJECT FUNCTION**						
Content	6	1	_	0.99	–	–
Spatial	6	0.99	0.01	0.91	−7.41	<0.001
Temporal	6	1	–	0.96	–	–
Other	6	1	–	0.98	–	–
Object use	6	0.99	0.01	0.94	−4.63	0.006
Object identification	6	0.97	0.02	0.83	−6.48	0.001
Identifies function	6	0.98	0.02	0.47	−23.61	<0.001

*Control participants (*n*), mean control proportion correct (Mean), control standard deviation (SD), Case AA’s proportion correct (Case AA’s scores) and *t*- and *p*-scores when Case AA was asked to produce action from verbal command, imitate action, and use objects*.

#### Pantomime from verbal command: intransitive actions

In contrast to his performance with transitive actions, Case AA was at ceiling for pantomiming the content and spatial properties of intransitive actions (15/15, 100%, for each). He committed one temporal (14.7/15, 98%) and one “other” error (14.7/15, 98%), respectively.

#### Imitation: transitive actions

Collapsing over all error types, Case AA was impaired relative to controls (13.7/15, 91%; *p* < 0.001; for all results see Table 2). The analysis by error type indicated that Case AA was similar to controls for content-related properties of the gestures he imitated (14.9/15, 99%). Spatial properties for imitated transitive pantomimes were impaired (11.6/15, 77%, *p* < 0.001), as well as temporal aspects of transitive imitations (14.3/15, 95%, *p* < 0.05). Case AA was at ceiling for other properties of the actions he imitated (15/15, 100%).

#### Imitation: intransitive actions

Case AA was at ceiling or similar to controls when imitating intransitive pantomimes. The spatial and temporal aspects of Case AA’s pantomime imitations were between 98–99% (14.8/15–14.9/15), and the content of his imitation was at ceiling (15/15; for all results see Table 2).

#### Tactile recognition, object use, and knowledge of object function

While keeping his eyes closed, Case AA was asked to identify objects from tactile exploration. An object was placed in front of him on a soft (i.e., noiseless) surface and he used his left hand to feel the object. If Case AA was able to identify the object he was asked to open his eyes. If Case AA was not able to identify the object with his eyes closed he was allowed to open his eyes in order to identify the object (however, the trial was scored as a 0 if Case AA was not able to identify the object with his eyes closed). Case AA was then asked to describe the function of the object in his hand, and to show how to use the object. Case AA’s ability to name objects from tactile feedback was worse than control participants (12.5/15, 83%, *p* < 0.01). Case AA’s ability to explain the function of tools was severely impaired with respect to control performance (7.1/15, 47%, *p* < 0.001).

The content of Case AA’s demonstrations of object use was similar to control participants (14.9/15, 99%), and Case AA was also similar to controls with respect to “other” properties of object use (14.8/15, 98%). However, as was the case for the pantomiming tests (see above), Case AA exhibited an impairment for the spatial (13.7/15, 91%, *p* < 0.001), and a mild impairment with the temporal, aspects of the produced actions (14.4/15, 96%; for all results see Table 2).

### Discussion

When Case AA was asked to judge if an observed action was familiar he was at ceiling; furthermore, when asked to match object names with a visually presented transitive pantomime he was not different than control participants. In contrast to his normal performance for action recognition, Case AA presented with impairments for action production: spatial properties of the transitive gestures Case AA imitated or produced from verbal command were impaired relative to control participants. In addition, when pantomiming from verbal command, Case AA committed “other” errors, as he would sporadically forget how to pantomime an object’s use. The temporal aspects of Case AA’s imitations and pantomimes from verbal command were also impaired, albeit less severely, as his accuracy was always in the mid-nineties, and statistically different due to small standard deviations among control participants^3^.

Note the dissociation in performance between transitive and intransitive gestures: Case AA was a ceiling or within control range when imitating and pantomiming from command intransitive gestures. This finding rules out limb weakness, confusion, or an inability to carry out the task as the cause of his difficulties with transitive actions. On the basis of the dissociation between imitating transitive and intransitive gestures it has been argued that there may be separate mechanisms that process transitive and intransitive actions (e.g., see Rumiati and Tessari, 2002; Tessari et al., 2007). Alternatively, transitive gestures may be harder to produce rather than processed by discrete cognitive mechanisms (Carmo and Rumiati, 2009; Mozaz et al., 2009). However, the results from the control participants do not suggest that task difficulty modulated performance when pantomiming from verbal command or imitating transitive gestures.

The dichotomy within transitive action production (i.e., impaired spatial content, spared conceptual content) was observed over several testing sessions, spanning 5 months. Thus, the main theoretical motivation of this investigation was to characterize the extent to which Case AA’s action knowledge was impaired, and the degree to which object concepts were commensurately damaged. Embodied cognition theories, as discussed in the Introduction, predict that conceptual analysis of tools necessarily requires retrieval of motor information necessary to use tools. Therefore it follows that the embodied cognition hypothesis would argue that conceptual knowledge for tools should be proportionately impaired in this individual.

### Experimental study III: Action-related object knowledge

#### Matching objects by function

A matching by function task was created using the same 15 objects in the Action Production tasks. On every trial Case AA was visually presented with pictures in a triad of three objects and was asked to decide which object (to the left or right of fixation) shared similar functional properties as the (top) target object. For instance, a triad could consist of scissors, pliers, and knife (where scissors and knife are used to cut; See Buxbaum et al., 2000; Buxbaum and Saffran, 2002; see also Garcea and Mahon, 2012). Case AA was within control range when making decisions about object function (13/15, 87%, *p* = 0.32). This finding is in contrast to his spontaneous production of the function of objects when the objects were in his hand; however, recognition tasks are generally easier than production tasks, and so the production task may be a more sensitive measure of AA’s abilities. In addition, Case AA’s knowledge of object function (using the same objects from the action production battery) classically dissociated from his ability to pantomime object use from verbal command: despite the fact that Case AA was impaired for spatial properties of the actions he was asked to pantomime, his knowledge of those objects’ function (as measured with the matching by function task) remained relatively similar to controls.

#### Matching objects by identity

In order to ensure that Case AA had no difficulty visually recognizing the objects he had been asked to use, a matching by identity task was created. This task was identical in format and materials to the *Matching objects by Function* test, except Case AA was asked to decide which object shared the same identity as the target object (but using different exemplars of the 15 tools). Case AA was at ceiling (15/15, 100%, *p* = 0.80) when asked to match objects based on identity.

#### Object sound decision

On every trial Case AA was presented with two nouns and had to decide which of two objects made the louder sound when used. Case AA was within control range when judging which object made the louder sound when used (27/31, 87%, *p* = 0.85).

#### Declarative knowledge of tools

Multiple-choice questions about properties of tools were auditorily presented to Case AA and control participants (for original design see Moreaud et al., 1998). The four questions examined goal of use (e.g., is a hammer used to nail, separate, or cut objects?), function of use (is a hammer used to do office jobs, cook, or build?), manner of use (to use a hammer, must you pull, lean, or swing with it?), and context of use (do teachers, doctors, or carpenters use a hammer?). Case AA was impaired with respect to control participants when deciding the precise use of tools (7.1/15, 47%, *p* < 0.001), and motor knowledge of tool use (9/15, 60%, *p* < 0.05). Case AA was impaired with respect to control performance for function of use questions (11/15, 73%, control range, 15/15), and context of use questions (13.1/15, 87%, *p* < 0.05). Interestingly, while always worse than controls, Case AA’s ability to make decisions about contextual information of tools (e.g., is a spoon used by a chef, a painter, or a doctor) was spared (i.e., strongly dissociated) relative to his knowledge of precise tool use (e.g., is a hammer used by swinging, throwing, or dropping).

### Discussion

Despite Case AA’s poor performance with action production, his knowledge of action-related object properties remained relatively intact (see Table 3). His ability to match objects based on their functional properties was similar to controls, and he was at ceiling when asked to match those objects with other exemplars of those same objects. Additionally, Case AA’s knowledge of the relative loudness of the sound given off by an object when used was intact. The former finding (spared function knowledge) is an issue that has previously been discussed in the context of apraxia. For instance, Buxbaum and Saffran (2002) and Buxbaum et al. (2000) found that apraxic patients with impairments for naming tools were also impaired when making decisions about which two of three objects were manipulated similarly; interestingly, those authors found that apraxics were relatively spared when making similar decisions about which two of three objects shared functional properties.

**Table 3:** **Action-related object knowledge**.

	Control sample			Case AA’s score	Significance test	
	*n*	*Mean*	SD		*t*	*p*
Matching by function	6	0.89	0.07	0.87	−0.27	0.32
Matching by identity	6	0.94	0.05	1	1.11	0.80
Object sound decision	6	0.89	0.09	0.87	−0.21	0.85
**DECLARATIVE KNOWLEDGE OF TOOLS**						
Precise use	6	0.93	0.06	0.47	−7.10	0.001
Motor knowledge	6	0.93	0.08	0.60	−3.82	0.01
Functional use	6	1	–	0.73	–	–
Contextual use	6	0.98	0.03	0.87	−3.40	0.02

*Control participants (*n*), mean control proportion correct (Mean), control standard deviation (SD), Case AA’s proportion correct (Case AA’s scores) and *t*- and *p*-scores when Case AA made decisions about action-related object properties*.

Thus, the neuropsychological dissociations between impaired manipulation knowledge and (relatively) spared function knowledge suggest that these different object properties may be processed by separable systems (for further discussion, see Garcea and Mahon, 2012). The data from Case AA lend credence to that hypothesis: despite Case AA’s impaired action production ability, his knowledge of object function was similar to controls. In the next section we investigated the degree to which Case AA’s knowledge of non-action object properties was spared.

### Experimental study IV: Form-, and color-related object knowledge

#### Object size judgment

Case AA and control participants were asked to decide which of two visually presented printed words (denoting noun concepts) were larger. Objects were from living and non-living categories (e.g., Which is larger, a hammer or a piano?). Case AA was within control range when making size judgments about object concepts (41/45, 91%, *p* = 0.39).

#### Object color judgment

Thirty black and white line drawings of items with prototypical colors from the Snodgrass and Vanderwart (1980) corpus were presented with two color choices. Case AA and controls were asked to decide which color best matched the line drawing; Case AA’s object color matching was within control range (27/30, 90%, *p* = 0.27).

#### Definition naming

A spoken definition was presented for Case AA and controls to identify; target items came from multiple categories of the Snodgrass and Vanderwart (1980) picture naming battery (e.g., fruits, vegetables, animals, body parts, musical instruments, tools, clothing, and vehicles). Case AA was at ceiling for fruit definitions (9/9, 100%, *p* = 0.15), and was within control range for vegetable (9/10, 90%, *p* = 0.45) and vehicle definitions (7/9, 78%, *p* = 0.48). Furniture definitions were marginally impaired (6/10, 60%, *p* = 0.05), and animals (5/9, 56%, *p* < 0.01), body parts (7/10, 70%, *p* < 0.01), musical instruments (4/9, 44%, *p* < 0.01), and tools (1/6, 17%, *p* < 0.01) were significantly impaired relative to control participants.

### Discussion

Case AA’s non action-related knowledge of objects was further assessed with several matching and naming tests. Case AA was similar to controls when making judgments about object size and color. However, and potentially directly relevant to the theoretical focus of the investigation, the patient was impaired for definition naming of several categories of objects (including tools). However, given that his impairment was general it is not clear what the source of Case AA’s impairment was. The majority of Case AA’s incorrect responses were timeouts (i.e., he did not respond within 10 s or could not come up with a name; see Table 4 for results).

**Table 4:** **Form-,and color-related object knowledge**.

	Control sample			Case AA’s score	Significance test	
	*n*	Mean	SD		*t*	*p*
Object size judgment	6	0.93	0.02	0.91	−0.93	0.39
Object color judgment	6	0.94	0.03	0.90	−1.23	0.27
**DEFINITION NAMING**						
Animals	6	0.90	0.05	0.56	−6.30	0.001
Body Parts	6	0.98	0.04	0.70	−6.48	0.001
Fruits	6	0.80	0.11	1	1.68	0.15
Furniture	6	0.93	0.12	0.60	−2.55	0.05
Musical instruments	6	0.85	0.06	0.44	−6.34	0.001
Tools	6	0.92	0.14	0.17	−4.96	0.004
Vegetables	6	0.83	0.08	0.90	0.81	0.45
Vehicles	6	0.83	0.06	0.78	−0.77	0.48

*Control participants (*n*), mean control proportion correct (Mean), control standard deviation (SD), Case AA’s proportion correct (Case AA’s scores) and *t*- and *p*-scores when Case AA made decisions about form-, and color-related object properties*.

While it has been established that Case AA is impaired when producing actions associated with objects, his knowledge of action- and non action-related properties of objects was relatively spared. We thus took to explicitly measuring Case AA’s action knowledge with a battery of tests that required Case AA to name and match actions with their associated names and objects.

### Experimental study V: Naming and matching objects and actions

#### Naming objects and actions

***Objects: snodgrass and vanderwart picture stimuli***. Two-hundred and sixty black and white line drawings of animals, fruits, furniture, kitchen items, musical instruments, tools, vegetables, and vehicles were presented for Case AA to identify (Snodgrass and Vanderwart, 1980). The stimuli were randomly ordered and Case AA completed this naming test on three separate testing occasions. The first two sessions were separated by 1 week; the third session was administered 4 months after the second session. However, the three scores were averaged into a composite score that was tested against control values; this procedure did not change any of the effects associated with the three individual sessions.

On the Snodgrass and Vanderwart Picture Naming task, Case AA was within control range for all categories except insects and fruits (name agreement values from 42 participants were obtained from Appendix B, Table B1 in Snodgrass and Vanderwart, 1980 and are summarized in Table 5); Case AA was impaired for naming fruits (8/11, 73%, *p* = 0.05) and marginally impaired when naming insects (3.36/8, 42%, *p* = 0.06). His errors were marked by omissions (no response within 10 s) and semantically related responses (e.g., cricket → beetle).

**Table 5:** **Naming and matching objects and actions**.

Picture naming	Control sample			Case AA’s score	Significance test	
	*n*	Mean	SD		*t*	*p*
**SNODGRASS PICTURE NAMING**						
Animals	42	0.90	0.10	0.87	−0.30	0.77
Birds	42	0.85	0.10	0.73	−1.19	0.24
Body Parts	42	0.88	0.13	0.95	0.53	0.60
Clothing	42	0.89	0.14	0.85	−0.28	0.78
Fruits	42	0.91	0.09	0.73	−1.98	0.05
Furniture	42	0.82	0.22	0.73	−0.40	0.69
Insects	42	0.75	0.17	0.42	−1.92	0.06
Kitchen	42	0.85	0.18	0.88	0.17	0.87
Music	42	0.85	0.13	0.85	0	1
Other	42	0.87	0.14	0.82	−0.35	0.73
Tools	42	0.92	0.12	0.87	−0.41	0.68
Vegetables	42	0.83	0.15	0.72	−0.73	0.47
Vehicles	42	0.85	0.16	0.83	−0.12	0.90
**NAMING OF ACTIONS**						
Action identification	64	0.85	0.05	0.36	−9.72	<0.001
**MATCHING OBJECTS AND ACTIONS**						
Picture-word matching: objects	6	0.98	0.01	0.94	−3.70	0.01
Picture-word matching: actions	56	0.92	0.05	0.72	−3.77	<0.001
Kissing and dancing	6	0.91	0.06	0.83	−1.23	0.27
Pyramids and palm trees	6	0.89	0.05	0.79	−1.85	0.12

*Control participants (*n*), mean control proportion correct (Mean), control standard deviation (SD), Case AA’s proportion correct (Case AA’s scores) and *t*- and *p*-scores when Case AA named Snodgrass and Vanderwart stimuli, action photographs, matched objects and actions with words, and performed the Kissing and Dancing, and Pyramids and Palm Trees test. Control values for the Snodgrass and Vanderwart Picture Naming test were obtained from Snodgrass and Vanderwart (1980); control values for the Action Identification and Picture-Word Matching: Actions test were obtained from Kemmerer et al., 2012*.

It is known that visual and linguistic factors (e.g., visual complexity, lexical frequency, concept familiarity) may affect picture naming speed and accuracy. We did not seek to statistically control (e.g., through logistic regression) the influence of visual and linguistic factors that might co-vary by semantic category, as the pattern of his category dissociation was not of theoretical importance. In other words, if it is the case that visual complexity or concept familiarity could explain the difficulty that Case AA had with fruit and insects, this is not germane to the theoretical goal of the current study, because Case AA’s ability to name tools was not impaired with respect to control participants.

#### Actions: action identification

One-hundred pictures of actions were presented for Case AA to identify. On every trial a picture was presented and Case AA was asked to name the action occurring in the picture with a one-verb response (e.g., juggling; for original materials see Fiez and Tranel, 1997; Kemmerer et al., 2001, 2012). The Action Identification task was administered twice over the span of 2 months, and controls values (see Table 5) were obtained from Kemmerer et al. (2012). Once again, we collapsed both sessions into one score; the pattern of results did not change when considering each session separately. Case AA was severely impaired when identifying actions (36/100, 36%; *p* < 0.001); his errors were marked by omissions and naming the objects in the photographs rather than the actions (squirting → spray bottle). Case AA persisted in naming the objects rather than the actions even after (repeated) explicit instructions were given to name the action performed in the photograph.

### Matching objects and actions

#### Picture-word matching with objects

Sixty-four black and white line drawings from the Snodgrass and Vanderwart (1980) corpus were presented with a word below each picture; on each trial Case AA was asked to decide if the picture and word were the same. The foils (i.e., “no” trials) were systematically related to the pictures: foils could be phonologically related (e.g., picture: pear, word: pencil), semantically related (e.g., picture: mouse, word: swan), or not related (e.g., picture: lemon, word: vase) to the target picture. Case AA was impaired relative to controls (113/120, 94%, *p* < 0.05). Of the seven errors he committed, five were semantically related, one was phonologically related, and one was unrelated.

#### Picture-word matching with actions

Sixty-nine verbs were presented in the infinitive form at the top of the screen (e.g., running) with two pictures depicting actions below the verb (for control values see Table 5; see also Kemmerer et al., 2012); Case AA was asked to decide which picture best matched the verb. Case AA was impaired when asked to match verbs and action pictures (50/69, 72%, *p* < 0.001).

#### Kissing and dancing test

Three verbs were presented in a triangular format and Case AA was asked to identify which verb to the left or to the right of fixation was most associated to the central target (for the original design and materials see Bak and Hodges, 2003). Case AA’s performance was not different than control participants (43/52, 83%, *p* = 0.27).

#### Pyramids and palm trees

The Pyramids and Palm Trees test (PPT; Howard and Patterson, 1992) was administered to Case AA on two test sessions separated by 1 week. On the first visit Case AA completed the picture version, and on the second session Case AA completed the word version. Case AA was not different than control participants when making conceptual decisions for pictures (41/52, 79%, *p* = 0.12). While the word version of this experiment was not administered to control participants, Case AA’s accuracy with word stimuli was comparable to his accuracy with picture stimuli (38/52, 73%, χ^2^ < 1).

### Discussion

**Figure 3:**
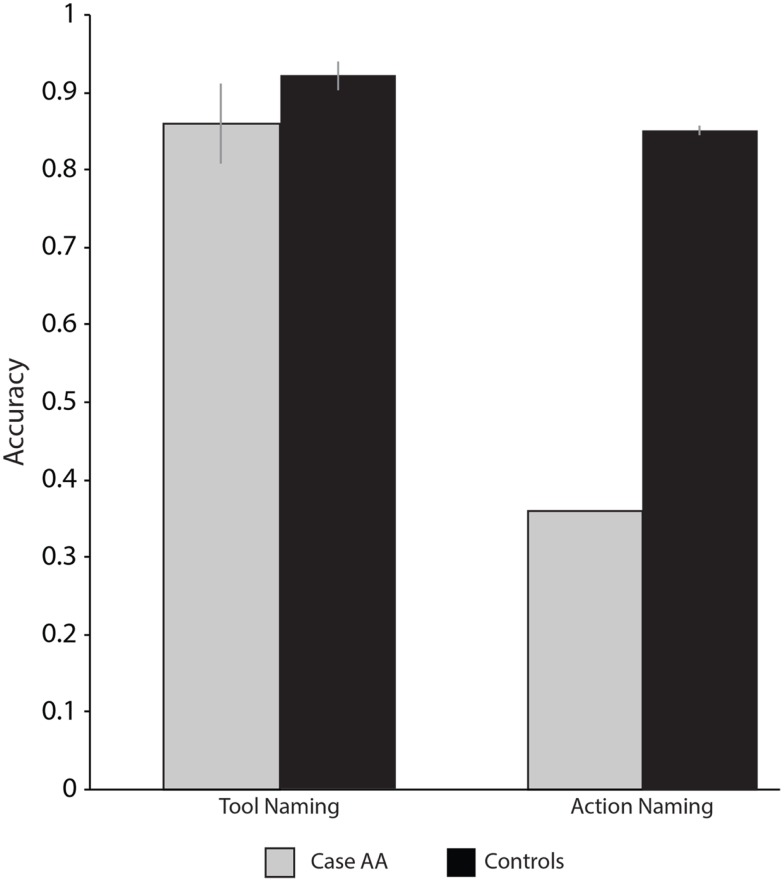
**Case AA and controls’ tool and action naming accuracy**.

When asked to identify black and white line drawings of objects, Case AA was largely unimpaired: Case AA showed marginal impairments for insects and fruit. All other categories of objects were within control range. It is particularly noteworthy that Case AA was within control range when naming the same tools that he showed impairments for when producing actions (for all naming results see Table 5; see also Figure 3). In contrast to his intact object naming ability, Case AA was impaired for naming actions. Case AA’s errors consisted of omissions (50%) and naming the objects in the pictures rather than the actions (39%). One possibility is that Case AA could have an impairment for verbs compared to nouns, rather than actions compared to objects (e.g., Caramazza and Hillis, 1991; Shapiro and Caramazza, 2003). A second (and not exclusive) possibility’s that Case AA had a semantic impairment for actions but not objects.

**Figure 4:**
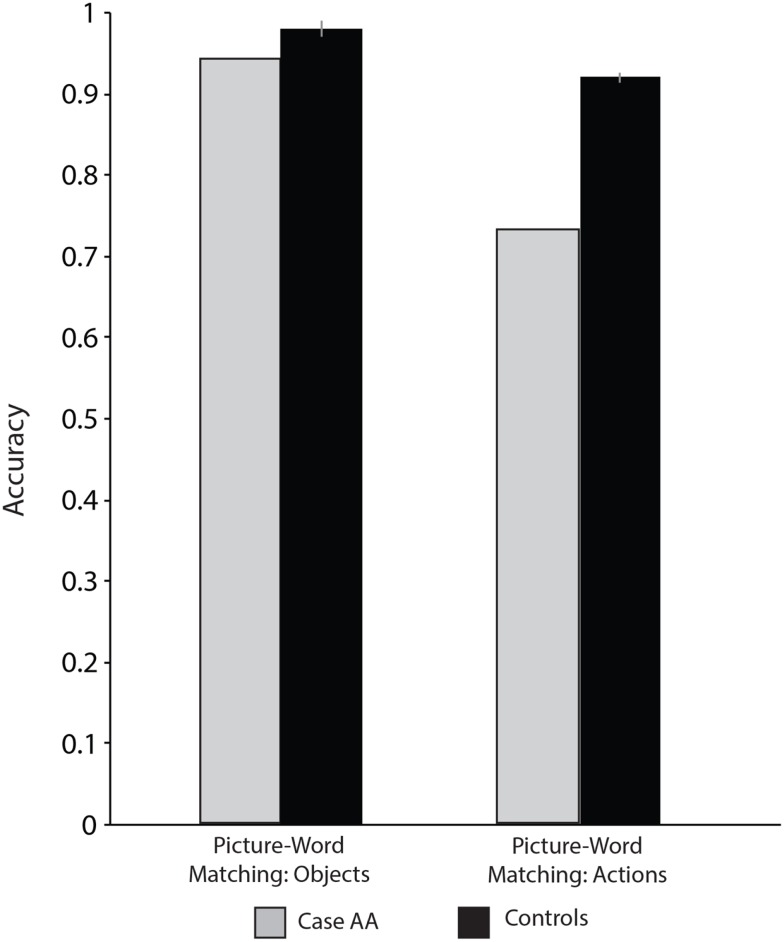
**Case AA and controls’ picture-word matching accuracy with actions and objects**.

It may be of note that while Case AA was severely impaired over a majority of the action tasks, he was not different than controls for the Kissing and Dancing test. While Case AA was impaired for matching pictures of both objects and actions to words, his ability to match pictures of objects to their corresponding words was overall less impaired than his ability to match action pictures and words (for all results see Table 5; see also Figure 4). In this context it is important to note that Case AA was equally as accurate when asked to read verbs and nouns (see *Linguistic Processing* in the Supplementary Materials). We therefore set out to further investigate the locus of Case AA’s impaired action knowledge, and to elucidate further whether this impairment affected Case AA’s object knowledge.

### Experimental study VI: Attribute knowledge of actions

Case AA completed the Attribute Knowledge of Actions battery (Kemmerer et al., 2012) on two separate occasions separated by 4 months. We collapsed session 1 and session 2 when calculating the modified *t*-test; this procedure had no effect on the magnitude of the difference between Case AA and control values. All control values can be found in Table 6 (obtained from Kemmerer et al., 2012).

**Table 6:** **Attribute knowledge of actions**.

	Control sample			Case AA’s score	Significance test	
	*n*	Mean	SD		*t*	*p*
Word attribute	56	0.95	0.04	0.68	−6.69	<0.001
Picture attribute	56	0.92	0.05	0.67	−4.96	<0.001
Word comparison	56	0.89	0.08	0.47	−5.20	<0.001
Picture comparison	56	0.84	0.08	0.33	−6.44	<0.001

*Control participants (*n*), mean control proportion correct (Mean), control standard deviation (SD), Case AA’s proportion correct (Case AA’s scores) and *t*- and *p*-scores when Case AA made attribute judgments of actions. All control values were obtained from Kemmerer et al., 2012*.

#### Word attribute test for actions

On every trial an attribute question (e.g., which would make the loudest noise?) and two verbs were presented (for control values see Table 6). Case AA was asked to decide which of the two verbs best satisfied the attribute question. Case AA was impaired relative to controls (42/62, 68%, *p* < 0.001). Interestingly, recall that when Case AA made similar decisions over object stimuli he was not different than control participants (see Object Sound Decision test).

#### Picture attribute test for actions

This test was identical to the Word Attribute Test but the stimuli were action photographs. Case AA was significantly different than controls (48/72, 67%, *p* < 0.001).

#### Word comparison test for actions

On every trial three verbs were presented and Case AA was asked to decide which two were most similar in meaning. Case AA was severely impaired and performed at chance levels (20.7/44, 47%, *p* < 0.001; chance cutoff: 66%).

#### Picture comparison test for actions

This was identical to the Word Comparison Test but the stimuli were action photographs. Case AA was at chance and significantly different than control participants (8/24, 33%, *p* < 0.001; chance cutoff: 71%).

### Discussion

**Figure 5:**
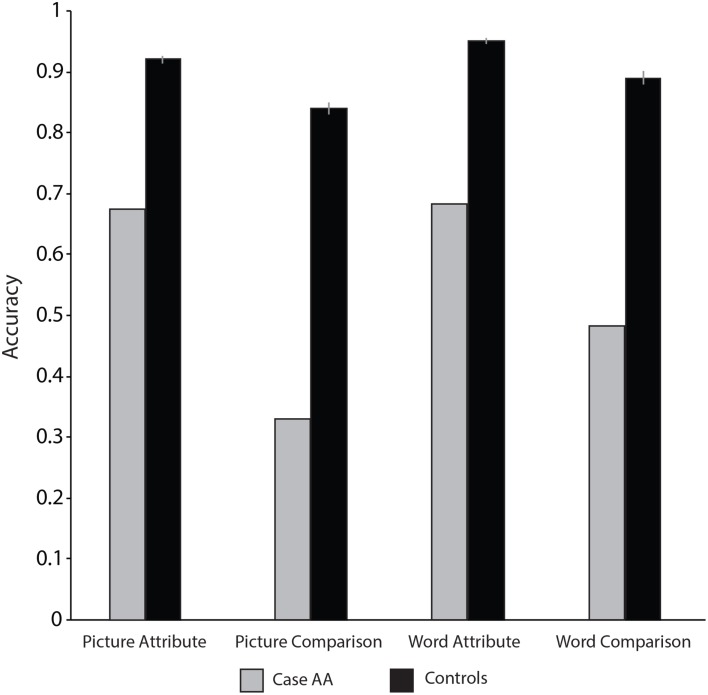
**Case AA and controls’ accuracy for attribute knowledge of actions**.

Case AA’s performance in the *Attribute Knowledge of Actions* battery provides more evidence that his impairment affected semantic information about actions. For instance, over a number of action property judgment tasks Case AA was at chance; those effects were consistent, and remained when Case AA was asked to perform the same action property judgment tasks 2 months later (see Table 6 for all results; see also Figure 5). Another example is the difference in performance when making loudness decisions with action and object stimuli: Case AA was impaired in the Word Attribute Test for Actions but was similar to controls when making loudness decisions in the Object Sound Decision test.

### Experimental STUDY VII: Semantic knowledge from non-linguistic auditory stimuli

In order to further investigate Case AA’s action knowledge impairment we developed several auditory sound-word matching experiments. Case AA and controls were presented with sounds of actions and objects, and were asked to match the sound that was presented with the appropriate action or object that it represents. This set of tests also permitted us to investigate the modality-independence of Case AA’s impairment for actions (i.e., if Case AA’s impairment was restricted to pictorial and lexical stimuli, or if Case AA’s impairment involved more generally the extraction of semantic information from action stimuli).

#### Limb- and mouth-related sound recognition

On every trial Case AA was presented with an action sound and two verbs, and was asked to match the sound with the appropriate action. The sounds were natural kinds (10 animal), limb-related (9 transitive, e.g., hammering; 10 intransitive, e.g., scratching one’s neck), and mouth-related (8 transitive, e.g., slurping soup; 10 intransitive, e.g., sneezing; for original experiment see Pazzaglia et al., 2008). In addition to the animal sounds, two non-biological noises (e.g., cooling fan buzzing) were included as filler items. The experiment was carried out twice, and the foils were manipulated such that there was an “easy” and “hard” version. The hard version was completed first, and the easy version was administered later that test session. The “hard” version was normed with age-matched controls, and was “hard” because the foils were effector-related to the targets and correct choices. The “easy” version contained foils that were unrelated to the correct answer. Case AA’s recognition of limb transitive (e.g., hammering; 9/14, 64%, *p* < 0.01) and mouth intransitive (7/10, 70%, *p* < 0.01) sounds were impaired in comparison to controls. Interestingly, mouth transitive discriminations were similar to controls (e.g., slurping from a straw; 7/8, 88%, *p* = 0.12). Case AA’s discrimination of limb intransitive action sounds (e.g., scratching neck), while not significantly different from control participants, was at chance (5/9, 56%, chance cutoff: 67%). In contrast to his poor performance with action stimuli, Case AA was not different than controls when discriminating animal sounds (9/10, 90%, *p* = 0.12).

#### Animal sound discrimination

On each trial two animal names were presented with an animal sound (e.g., cow mooing, dog barking) for Case AA to discriminate. Case AA was asked to match the correct animal name with the sound that was presented to him. His performance was within control range (16/20, 80%, *p* = 0.17).

#### Environmental sound discrimination

This test was identical in format to the *Animal Sound Discrimination* test: Case AA was asked to match the correct object name with the sound being presented. The sounds were comprised of human noises (e.g., yawning), tool noises (e.g., chainsaw), and natural sounds (e.g., ocean, rain); foils were semantically related to the correct answer choice. Case AA was mildly impaired relative to controls (12/15, 80%, *p* = 0.05). While his performance was mildly impaired, it is important to note that the three errors Case AA committed were not tool-related.

### Discussion

**Figure 6:**
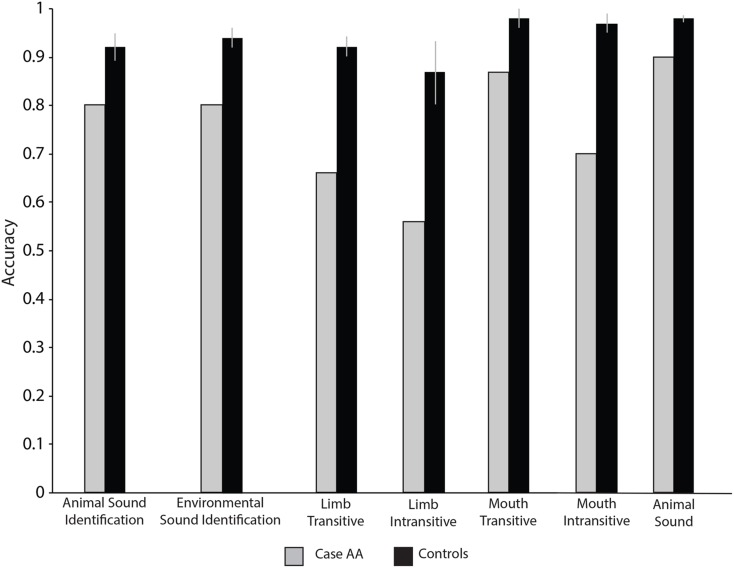
**Case AA and controls’ accuracy when discriminating object and action sounds**.

Case AA was consistently at chance or significantly different than controls when discriminating transitive and intransitive limb- and mouth-related sounds (see Table 7, and Figure 6). Pazzaglia et al. (2008) have shown that limb apraxia patients who were impaired for using objects were similarly impaired when making discriminations of limb-related sounds. Those authors also found that buccofacial apraxia patients who were impaired for producing gestures with their mouth, were impaired when making discriminations over mouth-related sounds. However, when discriminating animal sounds he was not different than controls, and when asked to discriminate bodily sounds and natural sounds his performance was only marginally impaired. These results help to clarify the boundary of Case AA’s impairment with action stimuli.

**Table 7:** **Semantic knowledge tested from non-linguistic auditory stimuli**.

	Control sample			Case AA’s score	Significance test	
	*n*	Mean	SD		*t*	*p*
Animal Sound Discrimination	6	0.92	0.07	0.80	−1.59	0.17
Environmental Sound Discrimination	6	0.94	0.05	0.80	–2.59	0.05
**LIMB- AND MOUTH-RELATED SOUND DISCRIMINATION**						
**Hard Version**						
Limb transitive	6	0.92	0.05	0.64	–5.19	0.004
Limb intransitive	6	0.87	0.16	0.56	–1.79	0.13
Mouth transitive	6	0.98	0.05	0.88	–1.85	0.12
Mouth intransitive	6	0.97	0.05	0.70	–5.00	0.004
Animals	6	0.98	0.04	0.90	–1.85	0.12
**Easy version**						
Limb transitive	–	–	–	0.79	–	–
Limb intransitive	–	–	–	0.56	–	–
Mouth transitive	–	–	–	0.88	–	–
Mouth intransitive	–	–	–	0.90	–	–
Animals	–	–	–	1	–	–

*Control participants (*n*), mean control proportion correct (Mean), control standard deviation (SD), Case AA’s proportion correct (Case AA’s scores) and *t*- and *p*-scores when Case AA made decisions about animal sounds, human/environmental sounds, and mouth-, limb-, and animal-related sounds*.

Although Case AA was impaired for limb- and mouth-related sounds, the pattern of performance is consistent with the results from other experiments: Case AA’s ability to extract semantic information from action stimuli is worse than object stimuli. This finding does not appear to depend on stimulus modality, as the dissociation between object and action semantics is preserved for linguistic, pictorial, and sound input.

## General Discussion

The theoretical objective of this study was to test the embodied cognition hypothesis of tool recognition with a detailed analysis of the dissociation between action and object knowledge in a 47-year-old individual who suffered a left CVA. Case AA presented with impairments for object-associated action production, both when pantomiming from verbal command, imitating action, and in actual object use. In addition, Case AA’s conceptual knowledge of action was moderately to severely impaired, and those impairments were stable across several months of testing. In contrast to his impaired performance with action production and action knowledge tests, Case AA’s object knowledge was relatively preserved: visual object recognition, object naming, and attribute judgments of several categories of object concepts were within control range.

As reviewed in the Introduction, a number of fMRI, TMS, and behavioral studies have been argued to support the embodied cognition hypothesis (Barsalou, 1999, 2008; Glenberg and Kaschak, 2002; Barsalou et al., 2003; Simmons and Barsalou, 2003; Zwaan, 2004; Gallese and Lakoff, 2005; Kiefer and Pulvermüller, 2012). At a general level, it is well established that the motor system is activated during tasks that do not require overt action or even the retrieval of action information (e.g., picture naming, word reading), when the meaning of stimuli implies action. The pattern of dissociated abilities we have reported in Case AA indicate that action information is not constitutive of manipulable object concepts. Here, ‘action information’ refers both to motor-relevant processes involved in actual object manipulation as well as more abstract semantic knowledge of actions. Here we step through the theoretical implications of the principal associations and dissociations in Case AA.

### 

#### Dissociation I: action production vs. action recognition

When asked to use actual objects, pantomime object use from verbal command, and imitate transitive gestures, Case AA committed spatial and temporal errors associated with the action (e.g., hand/finger misconfigurations). In contrast, his action recognition was largely or entirely preserved: He was able to make action decisions about and discriminate between meaningful gestures. Case AA was at ceiling or within control range when judging that intransitive actions were familiar, as well as matching transitive gestures with the appropriate tool. The observation of impaired action production in the context of spared action recognition has been observed in several other cases (Rapcsak et al., 1995; Rumiati et al., 2001; for the opposite dissociation see Rothi et al., 1986; Negri et al., 2007). That pattern of dissociation is problematic for the motor theory of action recognition (Gallese et al., 1996; Fadiga et al., 2002; Rizzolatti and Craighero, 2004; for critical reviews see Mahon and Caramazza, 2005; Hickok, 2009, 2010; Stasenko et al., in press).

One counterargument against this line of reasoning is that the foils used in the action recognition tasks with which Case AA was tested were foils of content. However, the types of errors that the patient made in action production were not errors of content, but rather spatio-temporal errors. In this context, it is important to note that not all of the tests involved foils of content (e.g., the test requiring recognition of actions as familiar or not). Nevertheless, future work with similar patients should systematically vary the nature of the foils to match the types of errors that the patient is making in production (see Rumiati et al., 2001 for such an approach).

#### Dissociation II: action vs. object knowledge

The observation that Case AA was unimpaired for naming objects but impaired for naming actions, and the associated impairments on tasks requiring non-verbal access to the semantics of actions, is problematic for the hypothesis that a necessary aspect of the meaning of manipulable objects involves action representations. For instance, according to the embodied cognition hypothesis of tool recognition, naming a visually presented picture of a hammer requires simulation of the motor processes that would be engaged in using that object. For instance, Case AA made spatio-temporal errors in transitive actions, but also had difficulty performing various matching tasks that did not require overt action production but instead required retrieval of semantic level information about actions. Similarly, multiple aspects of object knowledge were tested (e.g., object decision, picture naming, object color knowledge, object sound discrimination, matching objects by functional properties), and were relatively less impaired than action knowledge. Importantly, while Case AA’s performance was peppered with impairments at multiple levels of processing for actions, the various levels of object knowledge remained relatively preserved.

While it is clear that there is a privileged relationship between action representations and manipulable object identification, the neuropsychological data we and others have reported undermine the strong form of the embodied theory of tool recognition (Rothi et al., 1986; Ochipa et al., 1989; Rapcsak et al., 1995; Rumiati et al., 2001; Mahon et al., 2007; Negri et al., 2007; Papeo et al., 2010; for review see Mahon and Caramazza, 2005, 2008). One objection that may be raised about this conclusion is that a subtle impairment to object naming may have been missed with the coarse measure of accuracy. We thus set out to further elucidate Case AA’s ability to name manipulable objects with the more subtle measure of RT.

Magnie et al. (2003) conducted a norming study where undergraduate students were asked to rate items from the Snodgrass and Vanderwart corpus. Participants were asked to rate the ease with which they could pantomime an item’s use so that others could recognize the object that corresponds with that action (1 = no, 3 = unknown, 5 = yes). Magnie and colleagues ranked objects as ‘strongly manipulable’ if 80% of subjects rated the objects from 4 to 5; “strongly unmanipulable” objects were items for which 80% of participants rated from 1 to 2. Thus, it is possible to study the relationship between the naming performance and the manipulability of the items. An example of such an analysis is that of Wolk et al. (2005), who reported a patient with a disproportionate impairment for living things, and relatively less impaired performance for naming items high along the manipulability dimension. The authors argued that motor-based representation of objects with high manipulability indices insulated them from impairment. We have, in the context of our case, a clear opportunity to explore this very important prediction from almost the exact opposite direction: i.e., in a patient with apraxia of object use.

**Figure 7:**
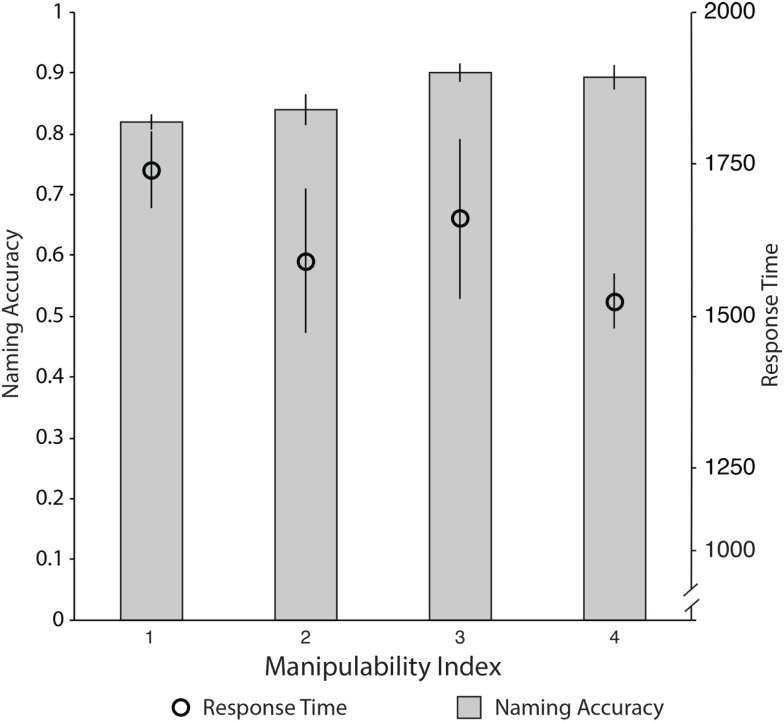
**Case AA’s naming accuracy and response time of Snodgrass and Vanderwart stimuli as a function of Manipulability Index from Magnie et al. (2003)**.

For simplicity, we calculated the average percent correct naming accuracy, and correct RT latencies for each item, and binned the data by manipulability index bins: (e.g., 1–2; 2–3; 3–4; 4–4.9) to derive a single naming accuracy, and a single RT latency for each discrete manipulability index (see Table 8; see also Appendix B in Wolk et al. (2005) for manipulability indices). Importantly, these are the same bins that Wolk and colleagues used. Case AA’s naming performance was positively correlated with the manipulability index, and the RTs were negatively correlated with manipulability index. That is, Case AA was more accurate and faster when naming manipulable objects with higher manipulability ratings (see Figure 7 and Table 8 for values).

**Table 8:** **Manipulability index naming analysis**.

	Case AA’s scores			
	PC	PC SD	RT	RT SD
Manipulability index 1	0.82	0.03	1741	125
Manipulability index 2	0.84	0.05	1591	237
Manipulability index 3	0.90	0.03	1660	262
Manipulability index 4	0.89	0.04	1526	91

*Mean Naming Proportion Correct (PC), Proportion Correct Standard Deviation (PC SD), Response Time (RT), and Response Time Standard Deviation (RT SD) of Snodgrass and Vanderwart Objects as a Function of Manipulability Index from Magnie et al. (2003)*.

While Case AA’s performance was (admittedly) weakly modulated by manipulability index, it is interesting to note that the trends in his naming accuracy and RTs mirror that of the patient reported by Wolk and colleagues. Thus, despite the fact that Case AA’s ability to produce actions was grossly impaired, his ability to name objects rated along the manipulability dimension goes against the prediction of the embodied cognition hypothesis: Case AA’s ability to name highly manipulable items should be impaired commensurate with his action production ability. However, we find the exact opposite pattern.

**Figure 8:**
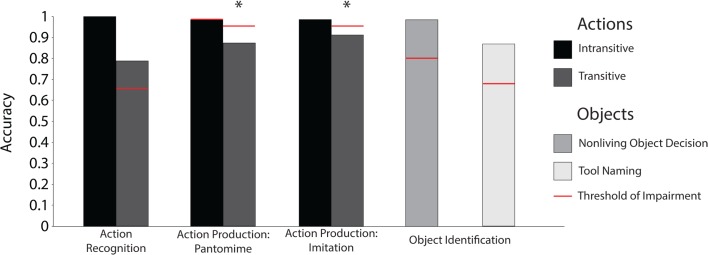
**Case AA’s principal dissociation between the ability to use and name manipulable objects**. * denotes a significant impairment relative to control participants. The threshold of impairment is plotted two standard deviations below control participants’ mean.

It should be noted that there is an association between action knowledge and action production: Case AA’s impairment in producing meaningful actions was correlated with his impairment for action knowledge. This suggests that damaging the ability to produce (and putatively simulate) meaningful action would have a deleterious effect on action semantics, which may rely, in part, on simulation; however, it is not clear that anyone would deny that action semantics is intimately related with motor-relevant information. Whether or not action knowledge is reducible to motor-relevant information is a separate question, and thus the question becomes whether action knowledge impairments dissociate from apraxia more generally. Critical, however, for present purposes, is that despite the fact that Case AA was impaired with action knowledge and action production, Case AA was able to name tools and match manipulable objects based on their functional properties (see Figure 8 for principal findings).

## Conclusions and Future Directions

We have argued that the available patient evidence, together with the new data that we have reported, are difficult to reconcile with strong forms of the embodied cognition hypothesis of manipulable object recognition. This conclusion raises the issue of what the implications are then of the range of findings that have been argued to support that hypothesis? We have argued elsewhere (Mahon and Caramazza, 2008; Garcea and Mahon, 2012) that inferences about the format of conceptual representations cannot be drawn without an articulated model of the dynamics of information exchange among sensory, motor, and conceptual representations. For instance, if it were the case that activation spreads between sensory-motor and conceptual levels of processing ahead of selection (i.e., cascading activation) the mere fact that motor processes are activated or engaged when viewing manipulable objects would have no implications for the format of the conceptual representation of that object.

While we have emphasized in the current case report a dissociation between impaired action knowledge and spared object knowledge, it is important to note that performance on action and object tests are correlated in large group level analyses. For instance, Buxbaum et al. (2005) (see also Negri et al., 2007) have observed that production and recognition of actions, or action knowledge and understanding of object concepts, tend to be correlated in large groups of patients (see also Pazzaglia et al., 2008). However, there is an asymmetry between associations and dissociations in their relevance to the hypothesis of embodied cognition: there are a number of possible explanations of associations. For instance, associations could arise from shared vasculature among the regions supporting functionally dissociable processes. One interesting possibility for future research is whether associations at the group level arise, in part, from disruptions in network function, caused either by damage to a hub or to white matter tracts. In contrast, it may be that selective loss of a knowledge type arises from lesions that largely spare the critical pathways mediating a broader network’s function, and/or from lesions that selectively affect a region that does not have hub-like properties. Patient-based investigations that combine the techniques and experimental paradigms that have been developed to study conceptual processing in healthy individuals have the power to open up new avenues for articulating a model of information exchange among sensory, motor, and conceptual processes, and the format of representations at those levels.

## Conflict of Interest Statement

The authors declare that the research was conducted in the absence of any commercial or financial relationships that could be construed as a potential conflict of interest.

## Supplementary Material

The Supplementary Material for this article can be found online at http://www.frontiersin.org/Human_Neuroscience/10.3389/fnhum.2013.00120/abstract

## Footnotes

^1^The modified *t*-test is computed by taking the difference between the patient’s score and the mean of the control sample, and dividing it by the product of the control sample’s standard deviation (SD) and the square root of the sample size (*N*), plus one, divided by the sample size. Thus, as the control sample size increases, the denominator decreases in size, and the *t*-score increases.

^2^In the text we report *t*- and *p*-scores associated with Case AA’s performance; see the Supplemental Online Materials for point and interval estimates, and effect size and effect size estimates for all tests that Case AA completed.

^3^We chose to score actions separately for content, spatial, temporal, and “other” action properties in order to have a sensitive measure to capture dissociations across different types of errors. It is important to note that this method underestimates the impairments Case AA had when producing transitive actions (e.g., Case AA scored a 13.95/15 for “other” errors, but those “other” errors were composed of Case AA not remembering how to pantomime object use from verbal command). In comparison, control participants never forgot how to pantomime object use from verbal command. This effect cannot be due to an impairment associated with pantomiming from verbal command in general, as Case AA was similar to controls when pantomiming intransitive actions from verbal command.
